# Molecular characterization of VIM1 isolates of *Pseudomonas aeruginosa* isolated from cases of sinusitis

**DOI:** 10.1186/1471-2334-14-S3-P62

**Published:** 2014-05-27

**Authors:** S Sasikala, S Niren Andrew, F Antony Irudhayaraj, T Sundararaj

**Affiliations:** 1 PG & Research Department of Microbiology & Biotechnology, Presidency College, Chennai, India; 2 Department of Microbiology, Madras Christian College, Chennai, India; 3 Department of Ear, Nose & Throat, Rajiv Gandhi Memorial Hospital, Chennai, India; 4 Jasmn Education & Research Foundation, Chennai-600096, India

## Background

Acquired metallo-β-lactamases (MBLs) are emerging resistance determinants in clinically relevant Gram negative species. These enzymes confer broad spectrum β lactam resistance, including resistance to carbapenems. Their potential for rapid and wide dissemination makes them of great concern.

## Methods

This study describes the molecular characterization of 15 *Pseudomonas aeruginosa* strains, isolated from endoscopic pus specimens, obtained from sinusitis cases. Susceptibility testing was performed by disk diffusion assay. The MIC of ceftazidime was determined by E test. Ceftazidime (CDM) - EDTA combined disc test was used to screen for class B β lactamase production. Plasmid DNA extraction was performed by alkali lysis method. The carriage of the MBL gene, *blaVIM* and IMP1 gene were screened by PCR. PCR products of 7 positive strains for MBL were sequenced and analysed using bioinformatics tools.

## Results

Metallo-beta-lactamase was present in 9 strains. These results correlated with the presence of VIM1 gene by PCR. IMP1 gene was absent in all the 15 strains. Plasmid was present in 10 strains only. The nucleotide and deduced protein sequences were analyzed with the software available over the Internet. The nucleotide sequences reported in the study has been submitted to the Genbank and accession numbers were obtained (KF975367, KF975368, KF975369, KF975370, KF975371, KF975372, KF975373). A Phylogenetic tree was constructed and it consisted of 2 clades. Both the clades had evolved from the common ancestor VIM-28.

## Conclusion

Direct detection of MBL genes may circumvent the problems of the phenotypic detection of carbapenemase producing microorganisms especially those exhibiting low carbapenem MICs.

